# Mothers’ perspectives on the delivery of childhood injury messages: a qualitative study from the growing up in Wales, environments for healthy living study (EHL)

**DOI:** 10.1186/1471-2458-13-806

**Published:** 2013-09-05

**Authors:** Ashrafunnesa Khanom, Rebecca A Hill, Sinead Brophy, Kelly Morgan, Frances Rapport, Ronan Lyons

**Affiliations:** 1 College of Medicine, Swansea University, Swansea SA2 8PP, Wales

**Keywords:** Childhood injury, Prevention, Message delivery, Neighbourhood deprivation, Migration, Health professional

## Abstract

**Background:**

Childhood injury is the second leading cause of death for infants aged 1–5 years in the United Kingdom (UK) and most unintentional injuries occur in the home. We explored mothers’ knowledge and awareness of child injury prevention and sought to discover mothers’ views about the best method of designing interventions to deliver appropriate child safety messages to prevent injury in the home.

**Methods:**

Qualitative study based on 21 semi-structured interviews with prospective mothers and mothers of young children. Mothers were selected according to neighbourhood deprivation status.

**Results:**

There was no difference in awareness of safety devices according to mothers’ deprivation status. Social networks were important in raising awareness and adherence to child safety advice. Mothers who were recent migrants had not always encountered safety messages or safety equipment commonly used in the UK. Mothers’ recommended that safety information should be basic and concise, and include both written and pictorial information and case studies focus on proactive preventive messages. Messages should be delivered both by mass media and suitably trained individuals and be timed to coincide with pregnancy and repeated at age appropriate stages of child development.

**Conclusions:**

The findings suggest that timely childhood injury-related risk messages should be delivered during pregnancy and in line with developmental milestones of the child, through a range of sources including social networks, mass media, face-to-face advice from health professionals and other suitably trained mothers. In addition information on the safe use of home appliances around children and use of child safety equipment should be targeted specifically at those who have recently migrated to the United Kingdom.

## Background

Early childhood is a critical time when injuries are most likely to happen. Unintentional child injury in the home costs the UK an estimated £25 billion a year [1] and represents a significant burden on public health care, local government, and to the families and individuals affected by it. Children below the age of four years are particularly at risk, with 2.5 injury-related deaths per 10,000 children in Europe, occurring annually [2].

Research shows that the burden of child injury is most prevalent in low income families [3] and factors in children’s physical and social environment determine their exposure to risk [4]. Parents can play a influential role in reducing their children’s exposure to risk by adopting good child care practices and by use of child safety devices in the home [4]. Factors associated with injury in the home include younger mothers [5], changing developmental stage of the child [6], socio-economic deprivation [3,7], inadequate use of safety equipment by parents or care givers [8] and the presence of siblings [9].

Parental lack of understanding or knowledge about the causes of accidents can be a significant barrier to the safety of young children in the home as found by a survey of 2088 parents of children aged 5 years in 14 European countries [2]. The authors conclude that parents would like to be better informed about the causes of child accidents and about actions they and society can take to reduce the risk of injury to children. Furthermore, several studies [10-12] have identified that inappropriate supervision by parents can cause injuries, even when parents are present. This calls into question parents’ level of knowledge and understanding in reducing hazardous practices in the home and use of safety equipment to prevent injuries.

Systematic reviews [13-16] on childhood injury prevention have identified numerous forms of intervention strategies. One approach widely advocated is the educational approach, which involves educating parents and children to modify their behaviour and encourage the use of safety equipment. However, there is always a inherent difference between gaining knowledge and implementing this knowledge into practice and can be dependent on the level of mothers’ education [17,18]. Changing the environment is another effective approach [7,19,20] when combined with infrastructural and institutional support [8,13,21,22], for example legislation to ensure safer environments (such as use of safe household appliances).

### Study aims

There have been few studies that have aimed to understand parents adherence to injury prevention messages [23] or to ascertain mothers’ views about the best method of designing interventions to deliver appropriate child safety messages to prevent injury in the home. This study invites mothers to talk about their current knowledge on childhood injury prevention, and asked for their recommendations on appropriate ways to design interventions aimed at delivering child safety messages. Thus, this study explores ways of improving mothers level of knowledge as a means of injury prevention.

## Methods

The mothers taking part in this qualitative study were chosen purposively from an existing birth cohort study “Growing up in Wales” [24] according to neighbourhood deprivation status. Mothers were recruited for the cohort study when they attended maternity appointments in hospitals and clinics within the Abertawe Bro Morgannwg (ABM) University NHS board and were interviewed either during pregnancy or when the child was 12 months old. The study protocol and consent forms were approved by the South East Wales Research Ethics Committee for Wales (09/WSE02/37). All mothers in the study were aged 16 years and older. They gave their own written informed consent to take part in the birth cohort study and again prior to each of the interviews. The investigation was conducted according to the principles of the Declaration of Helsinki [25] and upheld the ethical principles of Swansea University.

Our primary focus of this qualitative interview study was on neighbourhood deprivation as injury literature illustrates that childhood deaths from injury are influenced by socioeconomic factors irrespective of cause [3]. Therefore mothers selected for this study were categorised within the ‘deprived’ category ascertained by the Welsh Index of Multiple Deprivation (WIMD) [26], which includes deprivation based on: Income, housing, employment, access to services, education, health, community safety and physical environment.

We had little prior information on mothers’ views on how to disseminate information about child injury as the literature on this aspect of injury prevention is scant. As cohort mothers, participants who took part in this study had not previously been questioned on any matters relating to child safety, and their views had not been influenced by the research team prior to this study taking place.

Qualitative interviews were conducted according to guidelines for inductive qualitative research [27,28]. An inductive approach permits research findings to be influenced by both research objectives and raw data and could provide in-depth rich descriptions that could aid understanding of mothers’ perspectives on the delivery of childhood injury messages. We deemed child injury to be a sensitive issue, and as a consequence we selected a one-to-one semi-structured interview method rather than group data capture. Semi-structured interviews offered the opportunity to build rapport [29] with the interviewee to encourage mothers to frankly express views about their own child care practices and knowledge acquisition on how to prevent injuries, closely reflecting their actual lived experiences.

A semi-structured interview schedule was developed using a socio-ecological model that suggests the home environment and personal factors are likely to predict health behaviours [30,31]. Individual behaviours (attitudes and practices), personal feelings and individuals’ backgrounds were specifically examined during the interview to understand the context of current injury message provision. The interview questions assessed: 1) mothers’ views of existing childhood injury prevention messages and interventions, and 2) facilitators and barriers to using safety equipment in the home. Interviews took place in mothers’ homes (with the exception of one interview, which occurred in the workplace). During the interview mothers were questioned about their use of five safety devices in the home and their understanding of six hazardous practices which could lead to falls, poisoning, scalding and drowning (see the ' Safety devices and hazardous practices' subsection), as a starting point to promote discussion. Finally, free discussion [29] of personal experiences and ideas from parents was encouraged, in order to improve the delivery of childhood injury messages. The interview schedule evolved as the researcher aimed to explore emerging concepts.

### Safety devices and hazardous practices

Safety devices:

Safety gate/barrier

Thermostatic mixer valves

Fire guard

Child car seat

Cupboard restrictors

Hazardous practices:

Using baby walker

Leaving child in the bath, however briefly

Taking hot drinks (tea, coffee) while holding a baby

Not checking bath water temperature by hand

Hazards (medicines, cleaning products, knives, marbles, coins) in child's reach

The interviews were discontinued once researchers (AK and RAH) concluded that no new themes were likely to emerge (following the analysis of 21 transcripts). This was the point at which the researchers agreed data saturation had been achieved [32]. Theoretical saturation [32] was reached after 13 interview transcripts had been analysed and the remaining transcripts served purely to verify the findings. To ensure veracity of method, a senior qualitative researcher (FLR) was available during the analysis process to challenge and critique analytic outputs.

The analytical process (quotes, codes, categories and themes) followed the principles of inductive data analysis [27] where models and theoretical perspectives are informed by the interpretation of raw data. Interview transcripts were transcribed and inductive thematic analysis [33] was used by two researchers (AK and RAH) independently, to systematically draw out themes and categories from the interviews.

Initially interviews were read independently, to clarify key phrases or words and emerging concepts related to each of the three objectives: (1) mothers’ knowledge of child safety devices and practices, (2) facilitators and inhibitors of child safety practices, and (3) mothers’ recommendations for future child safety interventions to improve message delivery. After initial discussion between the two researchers, the first five transcripts were open-coded to identify quotes which could illustrate emerging concepts in relation to these categories. These open-codes and linked quotes then formed the basis of a codebook that was used to analyse the remaining transcripts. Throughout the coding process both researchers discussed the code-book and emerging categories and verified their findings through four paired analysis sessions, to ensure ‘inter-coder’ agreement [27,33]. Once the coding process was complete, codes were refined and clustered to allow sub-categories to be revealed.

## Results

Findings from the analysis of five interviews highlighted possible differences in outcome according to migration status between mothers. The sample of mothers was consequently further analysed to reflect these differences. Completion of data analysis produced two overarching themes (guided by the initial research aims) that pointed to the importance of mothers’ knowledge-acquisition and retention relating to childhood injury and facilitators and inhibitors of child safety. More detailed sub categories provided an in depth understanding of each overarching theme, these were awareness, access to information, social networks, birth order and personality and cognitive and physical development.

Twenty one interviews were conducted with mothers by the researcher (AK) over a period of three months. The average time for each interview was approximately 40 minutes and all interviews were tape recorded with mothers’ permission, transcribed verbatim and checked against the audio-recording. One interview was conducted in the participant’s mother language (Bengali) and was translated and transcribed by the researcher (AK).

The mothers’ ages ranged from 20 to 42 years of age (average age 30, standard deviation 6.5). The sample included 13 mothers from deprived neighbourhoods (DN) and eight from affluent neighbourhoods (AN). The diversity of the sample included mothers from Bangladesh, Pakistan, Wales, Poland, Romania, and Ghana. Five mothers identified themselves as recent arrivals to the UK (seven years or less) which included European and non-European migration. We found no definitive definition of a recent migrant. Several studies exploring the impact of recent migration to the UK have defined ‘recent’ as less than three years [34], five years [35], or 10 years [36] of residence in the UK. The United Nations [37] and the Office for National Statistics (UK) [38] defines long-term migrant as living in a country for 12 months or more. Such variation in the definition of recent suggests that, the acculturation into British culture and therefore knowledge and awareness of injury messages is also likely to vary. Our eventual findings did not indicate differences according to diversity of background or age amongst mothers. The importance of migration status was acknowledged during the process of interviewing, (the research team had not actively set out to identify mothers’ responses according to migration status). We also identified differences between first time mothers (who are faced with new challenges of caring for a baby), and mothers with one or more children (who have experience of caring for a child).

The results are presented around three main research objective: (1) mothers’ knowledge of child safety devices and practices, (2) facilitators and inhibitors of child safety practices, and (3) mothers’ recommendations for future child safety interventions to improve message delivery. Excerpts from the interview transcripts are shown in Table 1 to illustrate the results. Each mother has been assigned initials to denote deprivation status: DN: deprived neighbourhood and AN: affluent neighbourhood.

**Table 1:** Quotations from findings

	**Knowledge**
Purchasing equipment	“the first one was old one [car seat], like second-hand, we got it from eBay” (mother of one child: DN).
	“Obviously the higher up you go in price usually the safer they are [car seats]”, (mother of one child and pregnant: DN).
	“as long as everything is safety standard on the car seats [it’s ok to purchase item]”, (mother of two children and pregnant: DN).
	“I can’t really remember that much really of what she’s [health visitor] talked about”, (mother of two children: AN).
Health professionals advice	“Being given advice from someone who hasn’t got children never sits very well anyway… But if you have another parent who said what they did…then you’re going to listen”, (mother of two and pregnant: AN).
	“…You’d like to think a lot of [safety issues are] common sense, wouldn’t you? If you haven’t been around children at all and it’s all new, I suppose you do need to be told properly, don’t you?” (mother of one child: AN*).*
Alternative sources of information	“I have done the ESOL (English as a second language) course, they say where you can go and find these items [child safety devices]”, (pregnant mother: AN*).*
	*“*I like researching into what’s good for my child and what’s bad for my child and then make a decision myself”, (mother of one child: DN).
Social networks	“My auntie was telling me that one of her friends, when she had a baby, she was washing the back of the baby and she leaned him a bit too far forward and started drowning him. I thought if you can drown them that easily, you know, you don’t really want to leave them [in the bath unattended]” (mother one child: DN).
	**Factors affecting safe practice**
Birth order and personality	“… [My firstborn child] had quite cold baths for the first few months of his life because I was really worried the water would be too hot…[whereas] I just stick my hand in now. When I’m putting [my 14-month old] in, I just put her feet in first…See what she says (Laughs)” (mother of two children: AN).
	“…we used to have like a big book shelf in our lounge… he [older child] never even looked at it. Whereas, [younger child] would definitely have climbed up if she had the opportunity,” (mother of two children: AN).
	**Parental recommendations**
Verbal advice (one to one)	“I think going to the home of Asian ladies and talking to them, give them a leaflet [to look at] and explain to them. I think that would be the best” (pregnant mother: DN).
	“[have injury advice] in playgroups… because this morning we had people coming round who were brushing teeth… everybody sat round and listened, you know, because it’s for the kids” (mother of two children: DN).
Leaflets and books	“I quite like leaflets because you don’t always have the time to sit and talk…where you don’t have time to look at DVDs, videos or what not, but leaflets, you can just, when you’ve got a moment, have a quick look through, can’t you?” (mother of two children and pregnant: AN).
	“with leaflets and things, it’s just a waste of paper” (mother of two children and pregnant: DN).
	“when you start like a nursery they [should] give you like a little starter pack or something [on child injury prevention]” (mother of one child: DN).
Posters (in waiting rooms and nappy changing areas)	“because you sit and stare at [posters], quite often subconsciously you will have taken a lot of [the information] in” (mother of one child: AN).
Mass Media	“TV is probably the strongest point, there was an advert on TV not so long ago that was horrible… I suppose they need to put the information across to both parents, not just [the] mother …they need to target men more as well…, if they’re like watching the football, you know (mother of one child and pregnant: DN*).*
	“I text that St John’s Ambulance advert with the child choking [for an advice pack]” (mother of one child: DN).
Timing of interventions	“[Information should be given] at all stages… Because different things are relevant and will occur to you more at different stages. I think you need information not too long before it’s relevant… So whilst it’s good to read all this [safety information] before [having a baby], some of it will stick and some of it won’t” (mother of one child: AN).
	“…You don’t want to let [young children] harm themselves, you don’t want to wait until it is too late [to learn about child injury risks] and go ‘oh, I was supposed to get this, I was supposed to do this’, you know” (mother of one child: DN).

### Mothers’ knowledge of child safety devices and practices

#### ***Awareness of safety devices and hazardous practices***

There was no difference in the awareness of safety devices according to mothers’ deprivation status. Re-using car seats and other safety devices was common among mothers with more than one child, regardless of deprivation. Mothers perceived the purchase of safety equipment as costly so they sourced used items such as car seats and safety gates from friends, family and internet sites. They considered the merits of safety items according to ease of use and robustness. There were differences among some mothers from deprived areas who considered expensive items as better in terms of quality, while others relied on the British Standards Institution’s (BSI) Kitemark safety symbol in order to feel confident when purchasing less expensive equipment. There was less awareness of certain devices such as fire guards and home appliances (electric kettles presenting a risk of scalding), among mothers who were recent migrants to the UK. They reported having not used such items in their native countries.

Mothers were less aware of hazardous practices than they were of safety equipment. All mothers were mindful of the dangers of leaving hazardous objects within a child’s reach and especially of leaving a child unsupervised in a bath. However there was poor awareness of the need to check bath water temperature, of the dangers of taking hot drinks at the same time as holding a baby, and using a baby walker. These findings were the same irrespective of deprivation.

#### ***Source of information***

“Common sense”, social networks and health professionals, were main sources of information. Taking the common sense approach to child safety was notably absent from the narratives of recently migrant mothers. Instead their responses focused on previous personal experiences gained mainly through providing child care duties to family members and experiences of friends and families. Recently migrant mothers mentioned their awareness of child safety practices gained through their native education system. They also mentioned attending English for Speakers of Other Language (ESOL) classes in the UK, which had raised their awareness of the availability of child safety devices.

Mothers from deprived neighbourhoods also discussed the importance of social networks as an important source of child safety information. They discussed advice from family members and experiences within family contexts, and how these could shape mothers decision-making. Mothers expressed that previous experience of caring for children within a social network could help develop common sense and those who lacked child care experience were most likely to require additional support through health professionals.

Information about child safety was mainly provided to mothers by the health visitor in the months following the birth of the baby and, to a lesser extent, by the midwife during the hospital stay immediately after birth. Mothers living in deprived neighbourhoods received frequent home visits from health visitors compared to those living in more affluent areas. Some mothers were given a wide range of advice whilst others were given very little. Some welcomed the objective nature of such advice whilst others sought advice from friends and family members who had practical experience of child rearing. Mothers’ reported the problem of recalling verbal advice given by health visitors, especially if the advice was unrelated to their child’s level of development or was presented with a lot of other information.

Finally internet social forums were seen as useful sources of information and were used by mothers from both deprived and non-deprived neighbourhoods. Some mothers recognized the value of gaining information from a variety of sources to make informed decisions, illustrating a desire to be autonomous in their decision making.

### Facilitators and inhibitors of child safety

Social networks and British safety standards were major facilitators of child safety awareness. In the main, interviewees spoke more of inhibitors of their adherence to safety advice, including: the birth order and personality of the child, infant development, costs associated with safety products, and the use of alternative safety strategies.

#### ***Social networks***

Social networks proved vital in raising awareness of child safety and it emerged as a key facilitator of child safety awareness, and adherence to child safety advice. There was evidence of the influence of parental modelling of safety behaviour during their own childhood which encouraged mothers to continue these safe practices with their own children. Parental modelling was reported mostly by mothers living in deprived neighbourhoods.

#### ***Birth order and personality***

Mothers of first-born children were more likely to purchase safety packs including plug socket covers, door stops and cupboard restrictors, whereas mothers with previous children failed to mention this as they may already have these items. Mothers with older children adopted a more relaxed approach to assuring the safety of their youngest child(ren) such as running bath water and putting their older child’s foot in first to see if it is too hot. However, all mothers were cautious about leaving young children unsupervised with an older sibling.

Children’s “personality” was also referred to, and it was evident that the supervision of children was determined to a certain extent by the child’s nature and how “busy” they may be. Some children were considered quiet, whilst others were inquisitive and less risk averse and so required more attention.

#### ***Cognitive and physical development***

Mothers’ assumed that teaching a child about danger would protect them from injury. They assumed that children “need to learn” how to perform potentially risky tasks. Sometimes mothers delayed the purchase of safety equipment until they became aware of their child’s hazardous behaviour. Mothers based their use of safety equipment on their own personal assumptions of their child’s level of physical development and need. If they observed that their child was physically active and mobile then they would invest in stair gates and cupboard restrictors.

### Mothers’ recommendations for future child safety interventions

#### ***Format of message delivery***

The mothers’ narratives focused on the requirement to better inform parents of the long term effects of child injury risks and hazards in the home, in order to increase their knowledge and understanding of child safety. Several differences were identified in terms of preferences expressed by groups. New arrivals to the UK preferred one-to one advice with health professionals and leaflets with illustrations, to overcome cultural and language barriers.

Mothers from affluent backgrounds had a preference to learn about child safety matters from reading materials and by way of peer interaction, for example receiving verbal advice from playgroup leaders. On the other hand mothers from deprived neighbourhoods favoured more varied methods of intervention, such as the use of the media, posters, internet forums and one-to-one advice from health professionals. Suggesting dissemination of information may need to be slightly differentiated, depending on the extent of deprivation in a given area. All mothers’ recommended that overly-complex messages should be avoided and that information should be delivered in a concise format, in order to stimulate and maintain parental interest in its content, and to ensure that messages are fully understood.

#### ***Timing of interventions***

It was uniformly recommended that provision of information should begin during pregnancy and be repeated at appropriate times during infant physical development. Pregnancy was described as a time of “excitement” when prospective parents were more likely to choose to read, or have more time available to read, about parenthood and baby care. Mothers from deprived neighbourhoods made additional recommendations that pre-birth advice targeted to new or prospective parents should focus on informing parents about the uses and necessity of infant safety equipment, and provide a list of standard devices which are robust and yet inexpensive.

Providing child safety information in mothers’ maternity notes was recommended. It was suggested that information provided shortly after the birth of a child may be at a time when mothers were too busy to make changes around the home, with little time for reading, and when child safety advice could get lost among other information. Mothers’ suggested that information provision should be timed to occur “before you need it”. Interviewees were clear and unequivocal in their belief that information provided during pregnancy should be supplemented with further advice at developmentally-appropriate ages, to create an ongoing process of information provision. Information given could coincide immediately prior to a child’s development milestone, such as standing or walking, in order for effective prevention to be realised.

#### ***Delivery of interventions***

While it was believed that health professionals, and health visitors in particular, should play a part in delivering safety interventions, it was widely acknowledged that their heavy work demands can leave them little time to spend dispensing advice to families. Moreover, when a child reaches 12 months, mothers contact with a health visitor becomes less frequent and any visits that do occur tend to be clinic-based. As a result, health visitors may not always be available to provide advice on avoiding injuries at later stages of development. A novel recommendation was to use suitably trained individuals, who work on a day-to-day basis with families but need not be health professionals, to inform parents of child safety. Suggestions were made that mother and toddler groups could be called upon to reinforce child safety advice, and that an age appropriate safety pack with advice to parents could be provided when a child attends nursery.

## Discussion

Mothers in our study did have knowledge of common safety devices. However those who were recent arrivals in the UK were less likely to be aware of the dangers of household equipment not used in their country (e.g. kettles). Social networks were a source of information and helped encourage positive behaviours as mothers considered these behaviours as acceptable social norms. In addition, safety behaviours learned by mothers during childhood from their parents were practiced as adults, such as wearing car seat belts.

One important point of concern from the study was a lack of accessible advice regarding the use of pre-owned safety equipment. Mothers frequently mentioned re-using old safety gates and child car seats. The British Standards Institution (BSI) Kitemark [39] on safety products was one valuable source of information mothers from deprived neighbourhoods in particular found useful when purchasing toys and safety equipment with limited funds. They were reassured that pricing did not reflect the quality and safety of differentially priced devices.

Another area of concern was that mothers believed children were never too young to learn about injury risks, perpetuating the belief that children have the ability to remember safety rules and manage risky situations, and highlighting their lack of knowledge about child development [40]. This was regardless of the fact that mothers themselves had commented several times of their own difficulty in trying to remember safety messages which they had read, or which had been communicated to them by health professionals. Our study indicate that mothers were lacking in knowledge when it came to infant cognitive and physical development and as a consequence they delayed the purchase or installation of safety equipment before they perceived the need [41], thereby exposing their child to risks.

Mothers of first-born children were more likely to purchase multiple safety devices, whilst mothers with a number of children appeared to adopt a more relaxed approach to child safety. The increased confidence and belief in their own parenting skills could possibly reduce mothers’ adherence to safety practices. We also found mothers frequently invoking the notion of common sense as a means to injury prevention, but at the same time some mothers acknowledged that common sense was not always adequate in keeping children safe from injury. A Canadian study uncovered in a self reported interview study of 121 mothers that mothers’ belief in “common sense” was one of the determinants of childhood injury [40].

Our findings demonstrate that recently migrant families may lack awareness of commonly used household appliances, such as kettles, which might pose child safety risk. This finding is in accord with previous research which found that childhood injury rates in Hispanic immigrant families in the US were higher in those families where the caregiver had been resident in the host country for less than five years [42]. Other research has shown that close social networks can protect immigrant children from injury, due to the nature of sharing child care within families [41-44]. Coupled with the importance of the role of family members in raising awareness of child injury risks, (that was particularly common among mothers from deprived neighbourhoods [45]), our findings suggest that strengthening social networks could be a valuable strategy in injury prevention.

Injury messages can play a significant role in reducing injury. A survey of 900 parents with children ≤6 years of age indicated that adherence to safety messages by parents reduced home injury incidents by 36 percent [23]. Mothers in our study recommended that injury messages should be appropriate to the developmental age of the child, concise and logical to aid memory recall. Suggestions were made to place child safety information within advertising breaks in football matches to capture the attention of fathers, or within storylines of popular television dramas. It was recommended that injury messages should be communicated by influential and appropriately trained individuals and reinforced in a variety of settings, such as hospitals and clinic waiting rooms, child care and community venues, supermarkets, and nappy-changing areas. Case studies, written leaflets, books, social media and help-lines were also suggested. Our findings are supported by Aldoory et al. [46] who undertook a literature review to find that multi-component, multichannel campaigns (mass media, printed sources, and interpersonal) are most effective in injury prevention campaigns. They suggest employing a mix of voices including both peer and authority figures to ensure the trustworthiness and acceptability of messages. They also argue that simple messages are far more effective than complex behaviour change advice.

Limitations: This study only examined the views of mothers. Fathers and other care providers’ views were not included and they may give very different responses. In addition, self reported responses to interview questions may have affected the accuracy of our data and therefore there may be information bias. Despite these limitations the strength of our study arises from the inclusion of views from mothers of differing neighbourhood deprivation status, social background and migration status (which later emerged from the data). There have been very few studies in the UK which have documented home injury according to such diverse range of social background [47,48], and those that have done so have not categorised families in terms of migration status. Finally during the course of the interviews we identified that fathers have a role in preventing injury-related risks in the home. Possible future studies could interview mothers and fathers together, and move away from a polarised view of mothers as the only caregiver who is responsible for reducing childhood injury. This would truly allow for a socio-ecological multi-level [31] understanding of the issues around injury prevention and strengthen injury prevention strategies.

## Conclusion

This study found that social networks are an important source of child safety information. Injury messages should be delivered in an variety of formats with relevant age appropriate advice. Information should help parents understand child physical and cognitive development to reduce reliance on common sense. Finally information should be targeted at those mothers who are recent arrivals to the UK and may not have experienced the risks associated with household equipment used in the UK. We recommend that further studies in this area should include views from fathers.

## Competing interests

None of the authors have any financial or conflict of interest to declare.

## Authors’ contributions

Authors contributions include the following: Children in Wales (funders) conceived the original idea for the study. SB and AK developed the research proposal for funding. SB, AK, and RAH were responsible for the study design. FLR offered guidance on qualitative methodology and revising manuscript. AK was responsible for selecting and interviewing participants. AK and RAH carried out the qualitative analysis of interview data. AK supported by RAH were involved in drafting the manuscript. All the authors have read and approved the final manuscript.

## Pre-publication history

The pre-publication history for this paper can be accessed here:

http://www.biomedcentral.com/1471-2458/13/806/prepub
